# Factors associated with dietary practice and nutritional status of pregnant women in Dessie town, northeastern Ethiopia: a community-based cross-sectional study

**DOI:** 10.1186/s12884-019-2649-0

**Published:** 2019-12-23

**Authors:** Tona Zema Diddana

**Affiliations:** School of Nutrition, Food Science and Technology, Hawassa University, Hawassa, Ethiopia

**Keywords:** Dietary diversity, Nutritional status, Pregnant women, Ethiopia

## Abstract

**Background:**

Maternal undernutrition is highly prevalent in resource-poor settings. Hence, this study was intended to determine factors associated with the dietary practice and nutritional status of pregnant women in Dessie town, northeastern Ethiopia.

**Methods:**

Community-based cross-sectional study design was employed. Six hundred four (604) pregnant women have participated. A two-stage sampling method was applied to select participants. Socio-demographic and socio-economic data were collected using a structured interviewer-administered questionnaire. The dietary practice was measured using 13 dietary practice questions. Mid upper arm circumference (MUAC) was measured by standard nonstretchable MUAC tape. Data were entered into Epi-Info 7 and exported to SPSS version 20. Binary and multiple logistic regression analysis was conducted. Variables with *P* < 0.2 in bivariate analysis were entered for multiple logistic regression. At a 95% confidence interval, variable with 푃< 0.05 in multiple logistic regression analysis was considered statistically significant.

**Result:**

About 54.8% of the pregnant women had poor dietary practice and 19.5% were undernourished. First trimester of pregnancy (AOR = 0.46; 95% CI: 0.26, 0.80), no history of illness 2 weeks before data collection date (AOR = 0.42; 95% CI: 0.22, 0.80), poor perceived severity (AOR = 1.64; 95% CI: 1.15, 2.33), poor perceived benefits (AOR = 1.63; 95% CI: 1.14, 2.32) and poor self efficacy (AOR = 4.74; 95% CI: 2.94, 7.65) were significantly associated with poor dietary practice. Not attending antenatal care (ANC) (AOR = 3.46; 95% CI: 2.07, 5.78), illness (AOR = 1.93; 95% CI: 1.10, 3.5), poor dietary diversity (AOR = 5.92; 95% CI: 3.59, 9.76), poor nutrition knowledge (AOR = 3.03; 95% CI: 1.87, 4.92), poor dietary practice (AOR = 3.25; 95% CI: 1.91, 5.54) and poor perceived self efficacy (AOR = 5.59; 95% CI: 3.56, 8.79) were significantly associated (*P* < 0.05) with undernutrition.

**Conclusion:**

The dietary practice of pregnant women was suboptimal and nutritional status was relatively high. Being in the first trimester of pregnancy and no history illness were negatively associated while poorly perceived severity to malnutrition, poor perceived benefits, and poor self-efficacy were positively associated with the poor dietary practice. Not attending ANC, history of illness, poor dietary diversity, poor nutritional knowledge, poor dietary practice, poorly perceived self-efficacy were positively associated with undernutrition. Government, health extension workers and other concerned bodies should encourage pregnant women to attend ANC, promote health during pregnancy, strength and counsel to improve dietary diversity and practice of good nutrition. They should focus on the perceived belief of dietary behaviors.

## Background

Nutrition throughout the life cycle has a major impact on health. This is true for pregnant women as pregnancy is the most nutritionally demanding period in their life. Undernutrition and failure to receive essential nutrients in both quality and quantity during this physiologically demanding period would result in adverse pregnancy outcomes [1]. Undernourished mothers are more vulnerable to diseases, encounter more miscarriages and give birth to underweight children whose survival is at risk [2, 3]. Poor nutrition during pregnancy also contributes to the low weight gain of the mother, which is risk factors for the delivery of infants too small for gestational age leading to neonatal mortality and morbidity [4], failure to grow, slow cognitive development and chronic diseases in adulthood [5].

Globally, an estimated 15 million babies are born preterm [6] and about 20 million are born with low birth weight. More than 95% of these births are occurring in resource-poor countries [7]. Ethiopia is one of the developing nations with a high burden of maternal and child undernutrition, morbidity and mortality. The 2016 Ethiopian demographic and health survey (EDHS) report indicated that 22.4% of women’s reproductive ages (15–49 years) were undernourished and 29% were anemic at the national level. The prevalence of undernutrition was 22.9% in the Amhara region, the area where this study is conducted [8]. Pieces of evidence from local surveys in Ethiopia illustrated that undernutrition among pregnant women varies from 15.2–35.5% [9–11], and poor dietary practice women range from 39.3–66.1% [9, 12 and 13].

Inappropriate nutrition practice due to the wrong perception in combination with environmental and socioeconomic factors and infections are common causes of maternal undernutrition and mortality, low birth weight and intergrowth retardation [12]. A study conducted in northwestern Ethiopia indicated that attitude towards specific dietary habits, exposure to nutrition information; maternal education; income and nutritional knowledge were predictors of the dietary practice [13]. Another study from Bahir dar town, Ethiopia, showed that not taking iron supplements, lack of radio/television, husband’s income, maternal age and poor nutrition knowledge were factors for poor dietary practice [9]. Another study conducted in Shashemene district, Ethiopia, discovered that following antenatal care significantly associated with maternal dietary practice [12]. Similarly, age at first marriage and meal frequency [14], educational status [15], occupation of the head of household and religion [16], marital status [15, 16] was discovered as risk factors for undernutrition among pregnant women. Risk factors for maternal dietary practice and undernutrition might not be the same across different regions due to differences in socioeconomic characteristics, culture, ethnicity and geographical location. Furthermore, there is a literature gap on the dietary practice and nutritional status of pregnant women in the Dessie area. Hence, identifying community-based specific factors affecting maternal dietary practice and nutritional status is necessary and critical to design appropriate interventions. Hence, this study was conducted to determine factors associated with dietary practice and the nutritional status of pregnant women in Dessie town, Northeastern Ethiopia, 2017 G.C.

## Methods

### Description of the study area

This study was conducted in Dessie town, northeastern Ethiopia. The town is located in Amhara Regional State, northeastern Ethiopia. It is 401 Km away from the capital city, Addis Ababa and 475 Km away from the regional city, Bahirdar.

### Study design and study period

A community-based cross-sectional study was conducted in July 2017 G.C.

### Source population and study population

The source population of this study was pregnant women who were resided for at least 6 months in the town during the study period. The study population was pregnant women who were randomly selected from studied kebeles and included in this study.

### Sample size determination

The sample size was determined using a single population proportion formula indicated below. Prevalence of poor dietary practice among pregnant (*P* = 60.7%) in northwestern Ethiopia was taken to estimate sample size [9]. Margin of error (0.05), critical value at 95% confidence interval (Z1-ᾳ/2 = 1.96) and design effect (DE) = 1.5 was used. 10 percent (10%) non-response rate was added. Finally, 604 pregnant women were included in this study.

$$ n=\frac{{\left({Z}_{1-\frac{a}{2}}\right)}^2\ast p\ast \left(1-P\right)}{d^2} $$

### Sampling procedure and technique

A two-stage sampling technique was used. At the first stage, four sub-cities among 10 were selected using the lottery method. The lists of pregnant women (sampling frame) living in these four sub-cities were obtained. In the second stage, participants from each kebeles were selected proportionally by using a simple random sampling technique.

### Inclusion and exclusion criteria

All pregnant women who were lived for at least 6 months in the town and healthy (self-reported) were included. Pregnant women with disability (unable to speak) at the time of data collection were excluded.

### Variables

#### Dependent variables

Dietary practice and nutritional status of pregnant women.

#### Independent variables

Socio-economic and socio-demographic factors, obstetric and pregnancy-related factors, stage of pregnancy, nutrition knowledge, morbidity, dietary diversity, antenatal care (ANC) attendance and health belief model constructs (perceived severity, susceptibility, benefits, barriers, and self-efficacy).

### Data collection tools and procedures

Data were collected using a structured interviewer-administered questionnaire by eight trained nutrition professionals through the house to house visiting. Socio-economic and socio-demographic factors, health belief model constructs, nutrition knowledge, dietary practice, and anthropometric data were collected.

Sociodemographic and economic data were collected using a structured questionnaire adapted from the Ethiopian demographic and health survey EDHS (2016) [8]. Items of Health Belief Model constructs were measured using a five-point Likert scale (5 = strongly agree to 1 = strongly disagree). Then, the value of each Likert scale scored by participants for each question was summed and the mean was calculated.

Dietary diversity was collected using 10 food groups recommended by the food and agriculture organization of the United Nations (FAO). Based on FAO cut of points, dietary diversity is poor if less than five food groups consumed 24-h before the date of data collection; and good if a woman ate at least five food groups in the past 24-h before the date of data collection [17].

Nutrition knowledge was collected using 15 nutrition knowledge questions. Nutrition knowledge score was calculated by conducting factor analysis to reduce data and identify nutrition knowledge that explained most of the variance. Then, the nutrition knowledge responses that showed high variation in factor analysis were summed and the average (mean) was calculated.

The dietary practice was assessed using 13 dietary habit questions. The dietary practice score was obtained by summing each response given by participants. Participants were given score 1 if they correctly answer the question, favorable or healthy for dietary practice, and score 0, if they did not correctly answer the question, not favorable or healthy for dietary practice.

Nutritional status was determined by measuring middle upper arm circumference (MUAC). MUAC of the left arm was measured triplicate using a nonstretchable standard MUAC tape to the nearest 0.1 cm with no clothing on the arm. The average of triplicate measurement was taken. Pregnant women having MUAC< 23 cm were considered undernourished and ≥ 23 cm normal [18].

### Data quality assurance

A questionnaire was first prepared in English and then translated to the local language called Amharic. The training was given for data collectors and supervisors on methods of obtaining consent, study objectives, contents of the questionnaire, interviewing technique and MUAC measurement procedures. A pretest was conducted on 10% of the total sample size on pregnant women living in the area other than the study site. MUAC measurement was taken in triplicate to ensure accuracy. Overall data collection was monitored daily and the questionnaire was checked for completeness and consistency at the end of the data collection date.

### Data processing and analysis

The data were coded and entered into Epi-Info version 7. Then, it was exported to SPSS version 20.0 and checked for a missing value. The normality of data was checked by the Kolmogorov–Smirnov test. Multicollinearity was checked by variance inflation factors (VIF) test. Factor analysis was conducted to identify variables that explained high variability among nutrition knowledge responses. The result was summarized using frequency, mean, standard deviation and percentage. Bivariate logistic regression analysis was conducted to identify variables associated with dietary practice and undernutrition of pregnant women. One independent variable at a time entered to check associated with the dependent variable in bivariate analysis. Variables with a *p*-value of less than 0.2 in the bivariate logistic regression analysis were entered into a multiple logistic regression model to control confounders. The forward model selection method was employed in multiple logistic regression. At 95% confidence, variable with probability value (*p*-value) less than 0.05 was considered statistically significantly associated with dietary practice and nutritional status. The strength and direction of association were described using a crude odds ratio (COR) and adjusted odds ratio (AOR).

### Operational definitions

Good nutrition knowledge: women had good nutritional knowledge if she scored greater than or equal to mean.

Poor nutrition knowledge: women had poor nutritional knowledge if she scored less than mean.

Poor dietary practice: women had poor dietary practice if she scored less than 75% for dietary practice questions.

Good dietary practice: women had good dietary practice if she scored at least 75% for dietary practice questions [19, 20].

Poor perceived health belief (poorly perceived severity, susceptibility, benefits, and self-efficacy): a woman had poorly perceived health belief if she scored below mean.

Good perceived health belief (good perceived severity, susceptibility, exhibitions, and self-efficacy): a woman had good perceived health belief if she scored at least mean.

Poorly perceived barrier: a woman had poorly perceived barrier if she scored greater or equal to mean.

Good perceived barrier: a woman had good perceived barrier if she scored below the mean.

## Result

### Socio-demographic and economic characteristics of study participants

Socio-demographic and economic characteristics of the study participants were depicted in Table 1. Six hundred four (604) pregnant women were participated in this study making a response rate of 100%.

**Table 1:** Socio-demographic and socio-economic characteristics of the pregnant women in Dessie town, northeastern Ethiopia, 2017 G.C, (*N* = 604)

Variables	Frequency	Percent
Age of participants		
< 25 years^a^	182	30.1
26–35 years	264	43.7
> 35 years	158	26.2
Marital status		
Unmarried	170	28.1
Married	434	71.9
Ethnicity		
Amhara	325	56.8
Afar	122	20.2
Tigray	101	16.7
Oromo	56	9.3
Religion		
Orthodox	370	53.8
Muslim	154	25.5
Protestant	80	13.2
Head of house hold		
Father	377	62.4
Mother	181	30.0
Others	46	7.6
Educational status of the participants		
No formal education	182	30.1
Some education	422	69.9
Family income		
< 600ETB	183	30.3
601-1500ETB	224	37.1
> 1500ETB	197	32.6
Cash decision maker		
Father alone	188	31.1
Mother alone	134	22.2
Father and mother	282	46.7
Family size		
≤ 4 person	379	62.7
> 4 person	225	37.3
Household facilities		
No radio/television	86	14.2
Radio/television	518	85.8
Occupation of the head of household		
Not employed	148	24.5
Government employed	182	30.1
Pretty trader	89	14.7
Laborer	79	13.1
Others**	106	17.6

^a^The study did not include women below 18 years old since it is difficult to take consent from under 18 years; ** = non-governmental organization employed and students

### Pregnancy and nutrition-related characteristics of the study participants

Pregnancy and nutrition-related characteristics of the study participants are described in Table 2. About 348 (57.6%) of pregnant women were in the third trimester. About 411 (68.05%) had good nutritional knowledge, 428 (70.9%) practice good hygiene. On the other hand, 93 (15.4%) had been sick 2 weeks before the date of the survey.

**Table 2:** Pregnancy and nutrition related characteristics of the study participants in Dessie town, northeastern Ethiopia, 2017 G.C, (*N* = 604)

Variables	Frequency	Percentage
Stage of pregnancy		
First trimester	131	21.7
Second trimester	125	20.7
Third trimester	348	57.6
Have sources of nutrition information		
Yes	445	73.7
No	159	26.3
Attend antenatal care		
Yes	427	70.7
No	177	29.3
Take iron folic acid supplementation		
Yes	418	69.2
No	186	30.8
Reasons for not taking iron/folic acid supplement		
Bad taste	62	10.3
Ill complications	27	4.5
May harm child	25	4.1
Don’t know iron/folic acid supplements	27	4.5
Other reasons	27	7.5
Frequency of iron/folic acid supplement intake		
Always	308	51.0
Occasionally	110	18.2
Have good nutrition knowledge		
Yes	411	68.05
No	193	31.95
Practice good hygiene and sanitation		
Yes	428	70.9
No	176	29.1
Sick in past 2 weeks prior to date of survey		
Yes	93	15.4
No	511	84.6

### Women dietary diversity score

Nearly one-fourth (25.5%) of pregnant women had low dietary diversity and three fourth (74.5%) had good dietary diversity (Table 3). All (100%) of pregnant women were consumed cereal, roots and tubers 24 h prior to the time of data collection. Almost all (99%) were ate legumes 1 day before survey. Animal source foods such as meat/fish and poultry were consumed by nearly one fourth of participants and egg was consumed by 29.1% of pregnant women.

**Table 3:** Women dietary diversity of the pregnant women in Dessie town, northeastern Ethiopia, 2017G.C, (N = 604)

Food groups*	Frequency (percentage)
Cereals, white root and tubers, and plantains	100 (100.0)
Pulses/legumes	598 (99.0)
Nuts and seeds	69 (11.4)
Meat, fish and poultry (chicken)	158 (26.2)
Dairy and products	154 (25.5)
Egg	176 (29.1)
Dark green leafy vegetables	280 (46.4)
Other Vitamin A fruits and vegetables	145 (24.0)
Other fruits	290 (48.0)
Other vegetables	312 (51.6)
Women Dietary diversity	
Good (ate ≥5 food groups)	450 (74.5)
Low/Poor (ate < 5 food groups)	154 (25.5)

### Perceived health belief model constructs score

The result of perceived susceptibility to malnutrition, perceived severity to malnutrition, perceived benefits of practicing good nutrition, perceived barriers to practice good nutrition and self-efficacy to improve malnutrition and practice good nutrition is indicated in Table 4. About 78.3% of pregnant women perceived that they are susceptible to malnutrition and 60.3% perceived that malnutrition is severe for pregnant women and her fetus leading to health problems and death. About 83.9% of the participants perceived that practicing good nutrition has benefits in preventing malnutrition, 60.4% of participants had good perception towards barriers to practice good nutrition and prevent malnutrition and 81.5% had good perceived self-efficacy to practice good nutrition and prevent malnutrition.

**Table 4:** Perceived Health belief model constructs score of pregnant women in Dessie town, northeastern Ethiopia, 2017G.C, (N = 604)

Variables	Frequency	Percentage
Perceived susceptibility to malnutrition		
Good	473	78.3
Poor	131	21.7
Perceived severity to malnutrition		
Good	364	60.3
Poor	240	39.7
Perceived benefits of good nutrition		
Good	480	79.47
Poor	124	20.53
Perceived barriers to practice good nutrition		
Good	365	60.4
Poor	239	39.6
Perceived self efficacy to prevent malnutrition and practice good nutrition		
Good	492	81.5
Poor	112	18.5

### The dietary practice of pregnant women

The result indicated that 273 (45.2%) of pregnant women had good dietary practice while the rest 331 (54.8%) had poor dietary practice. Specific dietary practice related activates are depicted in Table 5.

**Table 5:** Frequency and percent of specific dietary practice related variables among pregnant women in Dessie town, northeastern Ethiopia, 2017 G.C, (N = 604)

Variables	Frequency	Percent
Crave food not normally consumed*		
Yes	278	46
Avoid any food items during current pregnancy*		
Yes	354	58.6
Reason of avoiding food items		
Personal dislike	179	29.6
Religion	39	6.5
Makes fetus big	17	2.8
Culture	15	2.5
Follow specific dietary regime*		
Yes	225	37.3
Meal frequency		
< 3 meals	284	47
3 meals	320	53
Eat snack between meals during current pregnancy*		
Yes	439	72.7
Skipping any meal during current pregnancy*		
Yes	131	21.7
Skipped Which meals		
Dinner	68	11.3
Breakfast	61	10.1
Lunch	2	0.3
Eat protein rich foods during current pregnancy^a^		
Yes	487	80.6
Habits of eating fresh fruits and vegetables^a^		
Yes	475	78.6
Use iodized salt^a^		
Yes	564	93.4
Time of adding salt into cooking food		
At the end of cooking	376	62.2
At the middle of cooking	76	12.6
At the beginning of cooking	146	24.2
Cook without any salt	6	1.0
Amount of fluid drunk per day		
Less than a liter	358	59.3
1–1.5 l	168	27.8
More than 1.5 l	78	12.9
Include the following in fluid		
Milk	92	15.2
Fruit juice	83	13.7
Soup	141	23.3
Herbal tea	288	47.8
Follow weight gain during current pregnancy		
Yes	281	46.5

^a^The remaining numbers (frequency) of participants belongs to “**No**” response

### Factors associated with the dietary practice

Factors associated with the dietary practice of pregnant women depicted in Table 6. Pregnant women at first trimester were nearly half less likely to have poor dietary practice as compared to the third trimester (AOR = 0.46; 95%CI = 0.26, 0.80). Women with no history of illness 2 weeks before the date of the survey were 0.42 times less likely to have poor dietary practice compared to their counterparts (AOR = 0.42; 95% CI: 0.22, 0.80). The poor dietary practice was 63% higher among women who perceived poorly to the severity of malnutrition compared to women who perceived malnutrition as severe (AOR = 1.64; 95% CI: 1.15, 2.33). Women having poor self-efficacy to practice good nutrition were 4.74 (AOR = 4.74; 95% CI: 2.94, 7.65) times more likely to practice poor nutrition than their counterparts. Similarly, the odds of practicing poorly was 63% higher (AOR = 1.63; 95% CI = 1.14, 2.32) among pregnant women who poorly perceiving benefits of practicing appropriate nutrition.

**Table 6:** Factors associated with dietary practice of pregnant women in Dessie town, northeastern Ethiopia, 2017 G.C, (N = 604)

Variables	Dietary practice (n)		COR (95% CI)	AOR (95% CI)	*p*-value
	Poor	Good			
Stage of pregnancy					
First trimester	54	77	0.53 (0.35, 0.79)	0.46 (0.26, 0.80)	0.016
Second trimester	78	47	1.24 (0.82, 1.89)	0.92 (0.53, 1.62)	0.78
Third trimester	119	149	1	1	
History of sickness					
No	266	245	0.47 (0.29, 0.75)	0.42 (0.22, 0.80)	0.01
Yes	65	28	1	1	
Perceived severity to malnutrition					
Poor	134	106	2.05 (1.47–2.85)	1.64 (1.15, 2.33)	0.006
Good	139	225	1	1	
Perceived benefits of good nutritional practice					
Poor	119	105	1.66 (1.19–2.32)	1.63 (1.14, 2.32)	0.007
Good	154	226	1	1	
Perceived self efficacy to control malnutrition					
Poor	85	27	5.09 (3.18–8.14)	4.74 (2.94, 7.65)	< 0.001
Good	188	304	1	1	

*AOR* Adjusted Odds Ratio, *COR* Crude Odds Ratio, *CI* Confidence Interval

### Factors associated with nutritional status of pregnant women

The result of factors associated with nutritional status revealed that 118 (19.5%) of pregnant women were undernourished (MUAC < 23 cm) and the rest 486 (80.5%) were normal (MUAC≥23 cm). Antenatal care (ANC) follow-up, history of illness, dietary diversity, nutritional knowledge, good dietary practice, and perceived self-efficacy to prevent malnutrition were showed significant association with undernutrition (Table 7). Accordingly, pregnant women who did not follow ANC during the current pregnancy were 3.46 times more likely to be undernourished than their counterparts (AOR = 3.46; 95% CI: 2.07, 5.78). Undernutrition was 93% higher in women who had a history of illness in the past 2 weeks before the survey date (AOR = 1.93; 95% CI: 1.10, 3.52). Women with poor dietary diversity (DDS < 5) were 5.92 times more likely to be undernourished (AOR = 5.92; 95% CI: 3.59, 9.76) compared to women having good dietary diversity. Undernutrition among women with poor nutritional knowledge and poor dietary practice was 3.03 (AOR = 3.03; 95% CI: 1.87, 4.92) and 3.25 (AOR = 3.25; 95% CI: 1.91, 5.54) times higher compared to their respective counterparts, respectively. Women having poor self-efficacy to practice appropriate nutrition and improve malnutrition were 5.59 times more likely to be undernourished (AOR = 5.59; 95% CI: 3.56, 8.79) compared to women with good perceived self-efficacy.

**Table 7:** Factors associated with nutritional status of pregnant women in Dessie town, northeastern Ethiopia, 2017 G.C, (N = 604)

Variables	Nutritional status		COR (95% CI)	AOR (95% CI)	*p*-value
	Undernourished	Normal			
Attend ANC					
No	69	108	4.93 (3.22, 7.53)	3.46 (2.07, 5.78)	< 0.001
Yes	49	378	1	1	
History of sickness					
Yes	33	60	2.75 (1.69, 4.47)	1.93 (1.10, 3.52)	0.033
No	85	426	1	1	
Dietary diversity					
Poor (< 5)	69	85	6.64 (4.30, 10.26)	5.92 (3.59, 9.76)	< 0.001
Good (≥5)	49	401	1	1	
Nutritional knowledge					
Poor	72	121	4.72 (3.09, 7.21)	3.03 (1.87, 4.92)	< 0.001
Good	46	365	1	1	
Dietary practice					
Poor	77	254	1.72 (1.13, 2.60)	3.25 (1.91, 5.54)	< 0.001
Good	41	232	1	1	
HBM (perceived self efficacy)					
Negative/Poor	52	60	5.59 (3.56, 8.79)	5.59 (3.56, 8.79)	< 0.001
Positive/good	66	426	1	1	

*AOR* Adjusted Odds Ratio, *COR* Crude Odds Ratio, *CI* Confidence Interval

## Discussion

Poor dietary practice and undernutrition among pregnant women have a major impact on fetus health and development. Nutrition of pregnant women should supply a sufficient amount of nutrients for mother, fetus and for successful lactation [21]. Various factors can influence dietary practice and nutritional status leading to poor pregnancy outcomes. The objective of this study was to determine factors associated with dietary practice and the nutritional status of pregnant women in Dessie town, northeastern Ethiopia. The overall dietary practice implies that maternal nutritional practice is sub-optimal in Ethiopia though health sectors developing different health and nutrition intervention programs.

The result revealed that 54.8% of the pregnant women had poor dietary practice and 19.5% were undernourished. The magnitude of poor dietary practice in this study is lower than 66.2% that was reported in the Oromiya region of western Ethiopia [13] and 59.9% reported in the Amhara region of northwestern Ethiopia [22]. Another cross-sectional study conducted by Amanuel and Tona (2018), in Bahirdar town, North West Ethiopia explored a higher prevalence (60.7%) of poor dietary practice [9]. This variation might be due to differences in a geographical area, agro-economic practices, the specific culture of a community, an individual’s dietary preference and seasonal variation in food production and consumption. On the other hand, agricultural food production practice varies across different geographic locations of Ethiopia. For instance, southern parts of Ethiopia produce mostly root and tuber crops, fruits and vegetables while northern parts of Ethiopia produce and depend on cereal crops. Ethiopia also has diverse religion followers. These factors are possibly caused variation in dietary practice.

Regarding specific dietary practices, about 47% of pregnant women had meal frequency less than three per day and 58.6% were avoided at least one food item during the current pregnancy. The main reason for avoiding food items was personal dislike, religion, belief that it makes the fetus big and culture. These reasons are in agreement with the study reported in northwestern Ethiopia [9]. The prevalence of pregnant women avoided food items in this study is higher than the study reported in southern Ethiopia where 21% of pregnant women avoided at least one food item [23]. The result of avoiding food items and meal frequency in this study is inconsistent with a study reported from the Oromiya region of Ethiopia where 35.8% of pregnant women were avoided at least one food item and 66.1% had meal frequency less than three times per day [13]. Another study from Addis Ababa, Ethiopia, illustrated that about 27.3% of pregnant women were avoided at least one food item during pregnancy [24]. This variation in the prevalence of good and poor dietary practices compared to another similar study might be duet individual difference in perception towards food and nutrient intake, household food security status and individual pregnancy-related complications. The difference might also be due to variation in socio-economic and socio-demographic factors; and cultural differences since research evidence suggests that dietary preferences could be influenced by cultural, social, economic and environmental determinants [24, 25].

In this study, 19.5% of pregnant women were undernourished (MUAC< 23 cm). The result is consistent with finding from Kenya where 19.3% of pregnant women were undernourished [26]. On the contrary, the result of the present study is higher than the study reported in china where 11.8% of pregnant women were undernourished [14]. Another study from the northern part of Ethiopia also indicated a lower prevalence (16.2%) compared to this study [9]. Similarly, a lower prevalence of undernutrition (15.2%) among pregnant women was explored in the Dale district of southern Ethiopia [10]. However, the prevalence of undernutrition in this study is lower than 32% reported in Bangladesh [27], and 35.5% reported in Boricha district of southern Ethiopia [11]. These variations might be attributed by the difference in stage of pregnancy, nutritional practice, a seasonal variation on food consumption and the use of different cut off points for mid-upper arm measurement in different studies.

History of illness 2 weeks before the date of data collection showed a negative association with poor dietary practice. This is unlikely but might be due to increased appetite and food requirements during the recovery period from illness. This might be made individuals follow the appropriate dietary practice. The finding of this study contradicts a study conducted in northern Ethiopia which indicated that the history of illness was positively associated with poor dietary practice [9]. This might be due to differences in types and severity of illness in two population groups. Being in the first trimester was negatively associated with the poor dietary practice. This might be due to the increased physiological demand for body nutrients in the first trimester made women practice good nutrition than practicing poor nutrition. The Health Belief Model (HBM) is one of the well-accepted models that refer to the key role of people’s beliefs. When people understand the level of risk that an unhealthy behavior poses and their susceptibility to the adverse consequences of their feelings, they become interested in methods that reduce their health and nutrition risks. In this study, poorly perceived severity of malnutrition, poor perceived benefits of practicing appropriate nutrition and poorly perceived self-efficacy to practice appropriate nutrition were associated with the poor dietary practice. This might be explained by that pregnant women did not fear for the severity of malnutrition, did not understand the benefits of practicing good nutrition for the fetus and herself, and unable to practice appropriate nutrition had a high chance to follow the poor dietary practice.

Not following antenatal care (ANC) during the current pregnancy was positively associated with undernutrition. This might be explained by when pregnant women attend ANC; they get nutrition information from health professionals and follow the healthy dietary practice. This is also in agreement with other finding such that women exposed to nutrition information were more likely to practice good dietary practice [23]. Following healthy nutrition further, improve nutrient intake and improve nutritional status. Women with a history of illness showed greater odds of being undernourished. This can be due to that there is a synergetic effect between malnutrition and illness such that illness increased energy and nutrient demand while decreasing food intake. This had resulted in women undernutrition. Dietary diversity also associated with nutritional status. This finding supported by a study conducted in Kenya in which dietary diversity was positively correlated with pregnant women’s nutritional status [26]. Similarly, a study in Pakistan illustrated that dietary diversity was significantly associated with a weight gain of pregnant women [28]. This might be because of as more food groups included in a daily diet, the greater the chances of meeting nutrient requirements by improving nutritional status [29]. On the other hand, different scholars demonstrated that dietary diversity is indeed associated with nutrient adequacy [30-32].

Poor nutritional knowledge and poor dietary practice were positively associated with maternal undernutrition. The odds of undernutrition among women with poor dietary knowledge and poor dietary practice were higher than their counterparts. This could be possibly due to that when a woman had poor nutritional knowledge, she became less focused on good dietary practice and not put it into practice. Besides, poor nutritional knowledge about basic nutrients and a balanced diet usually result in poor dietary practice leading to undernutrition. Not engaging in appropriate dietary practice contributes to low dietary diversity and nutrition intake leading undernutrition. Among dietary perceptions, women who perceived positive, confident and able to follow good nutritional practice (self-efficacy) had lower odds of undernutrition.

## Conclusion

The prevalence of poor dietary practice among pregnant women was 54.8% and undernutrition was 19.5% indicating that dietary practice is suboptimal and undernutrition is relatively high. Being first at trimester of pregnancy, history of illness, perceived severity to malnutrition, perceived benefits and self-efficacy were associated with the poor dietary practice. ANC attendance, history of illness, dietary diversity, poor nutritional knowledge, poor dietary practice and poorly perceived self-efficacy were significantly associated with undernutrition.

## Recommendations

Government, health extension workers and other concerned bodies should encourage pregnant women to attend ANC, prevent sickness and promote health during pregnancy, strength, and counsel to improve dietary diversity and practice good nutrition. Special focus should be given as a stage of pregnancy increase particularly in at third trimester. They should focus on the perceived belief of dietary behaviors particularly perceives the severity of pregnant women and fetuses to malnutrition, perceived benefits of practicing good nutrition and perceived self-efficacy to practice good nutrition and prevent malnutrition. This advice is for a similar situation in Ethiopia since the data in this study only apply to similar populations, but not places outside of Ethiopia or major urban areas.

## Limitation of the study

The current study did not evaluate food insecurity as a factor in maternal dietary practice and nutritional status though it is an underlying cause of malnutrition.

## Data Availability

The datasets used and analyzed for this study available from the corresponding author on reasonable request.

## Abbreviations

AOR: Adjusted Odds Ratio
CI: Confidence Interval
COR: Crude Odds Ratio
DDS: Dietary Diversity Score
ETB: Ethiopian Birr
G.C: Gregorian calendar
HBM: Health Belief Model
MUAC: Mid Upper Arm Circumference
